# Advances in Wearable Technologies for the In-Field Assessment of Biomechanical Risk

**DOI:** 10.3390/bioengineering12060632

**Published:** 2025-06-10

**Authors:** Micaela Porta, Massimiliano Pau

**Affiliations:** Department of Mechanical, Chemical and Materials Engineering, University of Cagliari, Via Marengo 2, 09123 Cagliari, Italy; massimiliano.pau@unica.it

The exceptional improvements that have characterized the technology of wearable devices for biomedical applications in recent decades have made it possible for researchers and practitioners to provide advanced solutions for the assessment of biomechanical and physiological variables in the present day, exploiting miniaturized, lightweight, and low-power-consumption devices at affordable costs. These devices, capable of collecting data onboard over extended periods of time, are well suited to continuous longitudinal monitoring in environments where other instrumental techniques may not be practical or effective. Due to all these favorable characteristics, and the fact that wearable sensors are mostly unobtrusive (and thus well accepted by workers), they have gained significant popularity in the ergonomics context, where they are fruitfully employed to assess the relevant biomechanical parameters associated with the development of work-related musculoskeletal disorders, thus overcoming most of the limitations typical of the observational methods (i.e., being time consuming, not suitable for dynamic tasks, and strongly dependent on the experience of the observer) routinely employed for ergonomic assessments. That said, the use of wearable technologies in real-world work environments is still far from being considered the “gold standard”, primarily due to the lack of standardization in setup procedures, measurement protocols, and data processing methods.

In this context, the aim of this Special Issue was to gather useful contributions for better delineating the benefits and drawbacks of different kinds of wearable technologies for the continuous assessment of biomechanical risk factors (e.g., posture, movement velocities, movement repetitions, loads) in actual working environments where the development of occupational musculoskeletal disorders represents a critical issue.

The review by Brambilla et al. [1], which summarized the results of studies on the assessment of fatigue, strain, and effort in real and simulated working scenarios, reported that, to date, most quantitative biomechanical analysis is lab-based, while questionnaires and scales still represent the preferred way to collect data “in field”. The authors pointed out that laboratory studies should be used as a benchmark to assess the physical state of the worker and identify the pathological changes associated with work-related musculoskeletal disorders. At the same time, further efforts should be made to transfer quantitative biomechanical assessments to workplaces to obtain data that are actually representative of the interaction between the workers and working environment.

Two studies proposed a method to continuously assess the Rapid Upper Limb Assessment (RULA) score using a motion capture system based on Inertial Measurement Units (IMUs). Feige et al. [2] applied this approach to a sample of dentists and endodontists while they performed canal treatments on a phantom head to investigate the existence of possible differences in the biomechanical load when different oral regions are treated. Similarly, Koskas and Vignais [3] continuously assessed the RULA score in hospital workers specialized in cleaning operating rooms. In this case, the automatized RULA method allowed for the identification of most critical subtasks associated with the highest risk of musculoskeletal disorder development.

Wearable sensors can also be employed to assess the effectiveness of supporting devices like exoskeletons. In this regard, Di Natali et al. [4], by combining data obtained from IMUs and sEMG, were able to discriminate between the different benefits associated with the use of two supportive exoskeletons during the performance of working tasks in the railway industry.

Finally, Slopeki et al. [5] proposed a very simple setup based on the use of a single IMU for the assessment of swimming performance in professional Paralympic swimmers. This approach demonstrated good performance as a tool for monitoring the number of movement repetitions performed over trials, training sessions, and weeks, allowing for in-pool workload monitoring to avoid overload and prevent the development of musculoskeletal injury.

In summary, the growing availability of wearable devices—offering increasingly powerful performance while becoming more user-friendly—has the potential to revolutionize how biomechanical aspects of movement are monitored across various settings. These advancements can play a crucial role in providing reliable, accurate, and robust data to effectively support biomechanical risk assessments.

## Data Availability

Not applicable.

## References

[1] Brambilla C., Lavit Nicora M., Storm F., Reni G., Malosio M., Scano A. (2023). Biomechanical Assessments of the Upper Limb for Determining Fatigue, Strain and Effort from the Laboratory to the Industrial Working Place: A Systematic Review. Bioengineering.

[2] Feige S., Holzgreve F., Fraeulin L., Maurer-Grubinger C., Betz W., Erbe C., Nienhaus A., Groneberg D.A., Ohlendorf D. (2024). Ergonomic Analysis of Dental Work in Different Oral Quadrants: A Motion Capture Preliminary Study among Endodontists. Bioengineering.

[3] Koskas D., Vignais N. (2024). Physical Ergonomic Assessment in Cleaning Hospital Operating Rooms Based on Inertial Measurement Units. Bioengineering.

[4] Di Natali C., Poliero T., Fanti V., Sposito M., Caldwell D.G. (2024). Dynamic and Static Assistive Strategies for a Tailored Occupational Back-Support Exoskeleton: Assessment on Real Tasks Carried Out by Railway Workers. Bioengineering.

[5] Slopecki M., Charbonneau M., Lavalliere J.-M., Cote J.N., Clement J. (2024). Validation of Automatically Quantified Swim Stroke Mechanics Using an Inertial Measurement Unit in Paralympic Athletes. Bioengineering.

